# Reliable chromatographic assay for measuring of indoleamine 2,3-dioxygenase 1 (IDO1) activity in human cancer cells

**DOI:** 10.1080/14756366.2021.1882451

**Published:** 2021-02-04

**Authors:** Ilona Sadok, Kamila Rachwał, Ilona Jonik, Magdalena Staniszewska

**Affiliations:** Faculty of Science and Health, Laboratory of Separation and Spectroscopic Method Applications, Centre for Interdisciplinary Research, The John Paul II Catholic University of Lublin, Lublin, Poland

**Keywords:** cancer cells, indoleamine 2,3-dioxygenase activity, kynurenine, tryptophan metabolites, glycation

## Abstract

The kynurenine pathway is the major tryptophan degradation routes generating bioactive compounds important in physiology and diseases. Depending on cell type it is initiated enzymatically by tryptophan-2,3-dioxygenase (TDO) or indoleamine-2,3-dioxygenase 1 and 2 (IDO1 and IDO2) to yield N-formylkynurenine as the precursor of further metabolites. Herein, we describe an accurate high-pressure liquid chromatography coupled with a diode array detector (HPLC-DAD) method to serve for IDO1 activity determination in human cancer cells cultured *in vitro*. Enzymatic activity was expressed as the rate of ʟ-kynurenine generation by 1 mg of proteins obtained from cancer cells. Our approach shows the limit of detection and limit of quantification at 12.9 and 43.0 nM Kyn, respectively. Applicability of this method was demonstrated in different cells (ovarian and breast cancer)exposed to various conditions and has successfully passed the validation process. This approach presents a useful model to study the role of kynurenine pathway in cancer biology.

## 1. Introduction

Indoleamine 2,3-dioxygenase 1 (IDO1) is a cytoplasmic, heme-containing enzyme that initiates of ʟ-tryptophan (ʟ-Trp) catabolism through the kynurenine pathway (KP). In addition to IDO1, another IDO isoform (IDO2) and primarily hepatic tryptophan-2,3-dioxygenase (TDO) might initiate the process of Trp oxidation into N-formylkynurenine (NFK) that is rapidly converted to a more stable KP metabolite - kynurenine (Kyn). Further degradation of Kyn results in the production of an array of downstream metabolites (collectively known as kynurenines) with immunoregulatory, antioxidant, or neurotoxic properties^1^^,^^2^. IDO1 being an immunosuppressive enzyme is expressed in various tissues and cells under both normal and pathological conditions, including cancer cells^3–5^. TDO and IDO2 also have been shown to be expressed in different cells within the cancer microenvironment and their contribution to Trp metabolism should be taken under consideration in cancer research^6^.

The primary finding showing IDO1 expression at the maternal–foetal interface and embryo implantation^7^ has started a series of studies on the role of IDO1 in several pathologies including neuropathology^8^, autoimmune disorders^9^, dermatological pathologies^10^, infections^11^, and cancer^12^. Although IDO1 expression and activity have been intensively studied in different diseases, many aspects of enzyme regulation still remain to be elucidated.

Numerous studies have linked IDO1 overexpression with poor survival in a variety of cancer patients including cervical^13^, endometrial^14^, colorectal^15^, and lung^16^ cancer. IDO1 activity associates with decreased activity of T-lymphocytes. T-reg activation and accumulation of immunosuppressive metabolites are identified as important microenvironmental factors in tumour survival. The opportunity of using IDO1 as a new prognostic marker or a molecular target in cancer treatment is recently considered in therapeutic approaches^12^. Human cancer-derived cell cultures represent an essential model used in cancer biology research. It enables to test the therapeutic efficacy of new anticancer compounds or identify new targets^17^.

Despite a lot that has been done in this field, challenges still exist in the field of development of robust biochemical assays for IDO1 activity measurements in the presence of potent inhibitors contain problematic functional groups^18^. IDO1 enzymatic activity is estimated by measuring the degree of tryptophan (substrate) degradation and kynurenine generation. The experimental approaches, including sample preparation, enzymatic reaction ingredients, or an end-point assay mode of measurement differ and might influence the obtained results^18^, therefore it becomes difficult to compare data generated by different laboratories. Some of the proposed methods for IDO1 activity assessment includes a system with SK-OV-3 human cancer cells stimulated by INFγ (known IDO1 stimulator)^19^ or yeast-based assay^20^. However, these also have some caveats related to unspecific cellular effects, toxicity, or incompatibility with human tissues^20^. Therefore, the development of an adequate IDO1 screening assay is still highly desired.

Depending on the approach, relative IDO1 activity is then expressed as a ratio of kynurenine-to-tryptophan (Kyn/Trp) concentration or is calculated for a gram of protein as an amount of kynurenine (mol/g) produced within a time frame (hour) that directly reflects activity. It is worth noticing that depending on the experimental system, Kyn and Trp can be quantified in different matrixes, like a solution of the purified enzyme, tissue homogenates, lysates of the cultured cells or the culturing medium from *in vitro* grown cells that contain secreted metabolites. Kynurenine amount generated in the assay can be determined by approaches like measuring the absorbance of the Kyn adduct in presence of *p*-dimethylaminobenzaldehyde (Ehrlich’s reagent)^21^, radioimmunoassay utilizing α,ʟ-[ring-2-^14^C]tryptophan^22^ or using the fluorescence-based^23–25^ assays. However, all of these methods have some limitations. The absorbance approach suffers from interferences caused by coloured compounds having absorption in the same wavelength and is incompatible with other compounds containing some functional groups (like ketones, aldehydes, hydrazines, indole, pyrroles, primary amines) that also react with Ehrlich’s reagent^18^^,^^26^. It might not be suitable to test some potent IDO1 inhibitors^27^ or interfere with other tryptophan metabolites (3-hydroxyanthranilic acid and 3-hydroxykynurenine)^28^. Much more sensitive radioimmunoassay requires working with costly radioactive tryptophan adducts^29^. Although the fluorescence measurements are rapid, it is affected by low pH that is most frequently used to stop an enzymatic reaction^23^. Furthermore, the method is prone to false-positive results caused by compounds with autofluorescence^24^. Using novel chemical probes - NFK-piperidine and NFK Green allowed for assay improvements ^24^^,^^29^. High-pressure liquid chromatography with UV type detector (HPLC-UV) is the most popular method for ʟ-Kyn determination in different biological samples^1^ and has also found application in IDO1 activity measurements in cells ^23^^,^^29^^,^^30^. HPLC systems provide high resolution of sample components, satisfactory accuracy, and reproducibility of the obtained results. Their versatility is due to the possibility of working with different modules, columns, and mobile phases. They are largely automated making basic runs easy to perform with minimal training. All the advantages make HPLC-UV methods attractive for identifying and quantifying chemical constituents in research and routine clinical studies.

While there are previous reports related to the application of different methods for IDO1 activity measurements in various types of cells, they usually focus on biological aspects of Trp catabolism or IDO1 properties and less on the development or adjustment of a determination method. Importantly, most of these methodologies were not fully validated and the obtained results might be inaccurate. Therefore, there is an unmet need to develop and validate a relatively simple but accurate protocol for IDO1 enzyme activity estimation in cellular models of cancer biology.

Addressing these limitations, we propose a method for direct IDO1 activity measurement in different human cancer cells based on the measurement of ʟ-Kyn production utilising a high-pressure liquid chromatography coupled with a diode array detector (HPLC-DAD). To achieve the goal, we optimised both the sample preparation step and chromatographic separation conditions. The protocol was adjusted using two representative cell lines derived from the human breast (MDA-MB-231) and ovarian (SK-OV-3) cancer with previously confirmed IDO1 expression^31^^,^^32^. The model cells provide universal conditions for IDO1 activity assay considered in cancer cell research. The presented methodology has been validated in terms of calibration, accuracy, precision, recovery, matrix effect, and selectivity. The accuracy of the method was confirmed by the ultrahigh-pressure liquid chromatography-tandem mass spectrometry (UHPLC-MS/MS) used as a reference method. Finally, the protocol has been successfully applied for IDO1 activity determination to show changes in enzyme activity depending on cancer cell type and treatment. We show IDO1 changes in presence of AGEs (advanced glycation end-products) – the panel of toxic adducts formed exogenously and in cells that have been earlier shown to induce inflammatory cytokines, like INF-*γ*^33^. In consequence, it can upregulate IDO1 expression in immune and cancer cells^34^^,^^35^. That makes AGEs and IDO1 the important players in the cancer microenvironment. We believe that using the proposed here optimised and validated HPLC-DAD method for IDO1 activity assessment will turn into an important analytical tool in future work on IDO1 role in the pathology of human cancer and testing potential enzyme inhibitors.

## 2. Materials and methods

### 2.1. Chemicals

Crystalline ʟ-Kynurenine (ʟ-Kyn, ≥ 98% HPLC), ʟ-Tryptophan (ʟ-Trp), 1-Methyl-ʟ-tryptophan (ʟ-1MT) were obtained from Sigma-Aldrich (St Louis, MO, USA). Stock solutions of ʟ-Kyn and ʟ-Trp (1 mM) were prepared by dissolving the reagents in dimethyl sulfoxide (DMSO) bought from Merck (Darmstadt, Germany). ʟ-1MT was dissolved in water containing 2% (v/v) of acetic acid (Merck, Darmstadt, Germany) to obtain 1 mM solution. All solutions were prepared in amber chromatographic vials and stored at −20 °C.

Cell culture was performed with foetal bovine serum (FBS), ʟ-glutamine and penicillin/streptomycin were purchased from PAN Biotech (Aidenbach, Germany). Trypsin, protease inhibitor cocktail, myoglobin from equine skeletal muscles (MB), and glycolaldehyde (GA) were obtained from Sigma-Aldrich (St Louis, MO, USA). Dulbecco’s Modified Eagle’s Medium (DMEM) containing 4.5 g/L of ᴅ-glucose were from the Institute of Immunology and Experimental Therapy PAS (Wroclaw, Poland). Phosphate buffered saline (PBS) solution was prepared by dissolving of 8 g of sodium chloride (Merck, Darmstadt, Germany), 0.2 g of potassium chloride (Sigma-Aldrich, St Louis, MO, USA), 1.44 g of sodium phosphate dibasic (Sigma-Aldrich, St Louis, MO, USA), potassium dihydrogen phosphate (Suprapur®, Merck, Darmstadt, Germany) in 1 L of water. Hydrochloric acid (37%) from Merck (Darmstadt, Germany) was used to adjusted pH to 7.4. Protease inhibitor cocktail was obtained from Sigma (St Louis, MO, USA). The reaction mixture for IDO1 assay was prepared using sodium ʟ-ascorbate, bovine liver catalase, methylene blue from Sigma-Aldrich (St Louis, MO, USA) and sodium phosphate buffer prepared using sodium dihydrogen phosphate and sodium phosphate dibasic purchased from Merck (Darmstadt, Germany) and Sigma-Aldrich (St Louis, MO, USA), respectively. Bradford reagent and bovine serum albumin (BSA) standards were purchased from Bio-Rad (München, Germany). The mobile phase for HPLC-DAD analysis was prepared using methanol (HPLC grade), ammonium acetate and glacial acetic acid (Suprapur^®^) purchased from Merck (Darmstadt, Germany). UHPLC-MS/MS analysis was carried using methanol (hypergrade, Merck, Darmstadt, Germany) and ammonium formate (for MS ≥ 99.0%, Sigma-Aldrich, St Louis, MO, USA).

A reaction mixture for the preparation of ʟ-Kyn working solutions and calibration standards contained 1.26 ml of PBS, 0.56 ml of 0.25 M sodium phosphate buffer pH 6.5, 0.28 ml of 0.1 mM methyl blue, 0.28 ml of 0.2 M sodium ʟ-ascorbate, 0.24 ml of 30% (w/v) trichloroacetic acid (TCA, Sigma-Aldrich, St Louis, MO, USA) and 0.42 ml of ultrapure water.

AGEs (MB-GA) were obtained by dissolving myoglobin (MB) and glycolaldehyde (GA) in PBS followed by incubation for 21 days at 37 °C. The unbound aldehyde was next removed by dialysis using an Amicon centrifugal system (Merck Millipore, Tullagreen, Ireland) and washing the filter with ultrapure water. The prepared MB-GA was lyophilized to determine the total weight of generated AGEs that were used for cell treatment.

### 2.2. Equipment

The cancer cells were cultured in the HERAcell 150i Cu incubator (Thermo Fisher Scientific, Schwerte, Germany). The optical microscope Olympus IX73 (with CellSens Dimension software for image analysis, Olympus) was used for cell monitoring and cell number was determined using a Bürker’s chamber.

Crystalline chemicals were weighed on the XP6 microbalance from Mettler Toledo (Schwerzenbach, Switzerland). Samples were centrifuged using 5804 and 5415 R Centrifuge purchased from Eppendorf (Hamburg, Germany) and incubated using a Stuart block heater (SGH130D/3, Bibby Scientific Limited, Stone, Staffordshire, ST15 USA). SevenMulti™ dual metre pH/conductivity equipped with the InLab^®^ Expert Pro electrode (Mettler Toledo, Schwerzenbach, Switzerland) was used for pH estimation. The absorbance measurements for total protein estimation were carried out using a multi-mode microplate reader Synergy 2 (Bio Tek Instruments Inc, Winooski, USA) operated with the Gen5 1.09 software. Ultrapure water used for chemicals and sample preparation was produced by a HydrolabPolska System (Staszyn, Poland).

Kynurenine content in cancer cell extracts was determined with an HPLC-DAD system on the 1200 series high-performance liquid chromatograph (Agilent Technologies, Santa Clara CA, USA) equipped with an autosampler (G1329A), quaternary pump (G1311A), degasser (G1322A) and diode array detector (DAD, G1316A). The separation was achieved using a Zorbax Eclipse Plus-C18 Rapid resolution HT 4.6 × 150 mm, 3.5 µm column connected with a Zorbax Eclipse Plus-C18 2.1 × 12.5 mm, 5 µm Narrow Bore Guard Column both bought from Agilent Technologies (Folsom, USA). Instrument control and data analysis were carried using Agilent ChemStation software v.B.04.02.

The reference UHPLC-MS/MS measurements were conducted using the 1290 Infinity series ultrahigh performance liquid chromatograph (UHPLC) coupled to the 6460 triple quadrupole mass-spectrometer (Agilent Technologies, Santa Clara CA, USA). The UHPLC system consisted of an autosampler (G4226A), binary pump (G4220A), column thermostat (G1316C), and DAD detector (G4212A). The mass spectrometer equipped with an Agilent Jet Stream ion source (G1958-65138) was operated in positive mode. Zorbax Eclipse Plus C18 rapid resolution HT 2.1 × 50 mm, 1.8 µm column and the Zorbax Eclipse Plus-C18 2.1 × 12.5 mm, 5 µm Narrow Bore Guard Column (Agilent Technologies, Folsom, USA) were used for analyte separation. Instrument control was carried using the Agilent MassHunter Workstation Data Acquisition software v.B.08.00. The data were processed by the Agilent MassHunter Quantitative and Qualitative Analysis software (v.B.07.00).

### 2.3. In vitro culture of cancer cells

Human breast carcinoma cell line MDA-MB-231 and human ovarian cancer cell line SK-OV-3 were from American Type Culture Collection (ATCC). The complete culture medium contained Dulbecco’s Modified Eagle Medium (DMEM) supplemented with 2 mM ʟ-glutamine, 10% (v/v) heat-inactivated foetal bovine serum (FBS), 100 IU/ml penicillin and 100 μg/ml streptomycin. Cells were grown in the incubator set at 37 °C, in high humidity conditions with 5% CO_2_. Petri dishes were used for culturing and cells were passaged at approximately 80% confluency. The cells were washed with PBS (5 ml) and detached from the Petri dish using trypsin (1 ml). Trypsin was stopped by addition of the complete culture medium (3 ml) and cells were collected into a Falcon tube, followed by counting using Bürker’s chamber and transferred in Eppendorf tube (1 × 10^6^ cells) for method optimisation. The experimental cells were seeded into 12-well plates (0.3 × 10^6^ cells per well) and allowed to attach overnight. Subsequently, fresh DMEM with 5% (v/v) FBS (control) or DMEM containing 100 µg/ml MB-GA was added to each well, and cells were treated for 48 h.

### 2.4. Sample preparation for measuring of IDO1 enzymatic activity

The cells from each well of the 12-well plates were collected by trypsinization and transferred into separate Eppendorf tubes. The cellular pellet was washed with PBS and after centrifugation (1100 × *g*, 5 min, 4 °C) was immediately subjected to further preparation steps for IDO1 activity estimation. The cells (kept on ice) were resuspended in 200 µl of PBS containing 2 µl of protease inhibitor cocktail (for 1 × 10^6^ cells), vortexed well and disrupted by 3 cycles of freezing at −20 °C and defrosting on ice. After centrifugation (14,000 × *g*, 5 min, 4 °C) the supernatant (containing cytosolic fraction) was collected for determination of protein content by the Bradford method^36^^,^^37^. Enzyme activity was assessed based on the modified methodology presented earlier^38^ using 90 µl of the cytosolic fraction obtained from the cell lysate that was immediately added into 150 µl of the IDO1 activity reaction mixture composed of 50 mM sodium phosphate buffer (pH 6.5), 20 mM ascorbic acid sodium salt, 200 µg/ml bovine liver catalase, 10 µM methylene blue, and 100 µM ʟ-Trp. The reaction mixture in case of control samples for inhibition studies contained as a substrate 0, 20, 100, 200 or 400 µM 1-methyl-ʟ-tryptophan (ʟ-1MT, an IDO1 inhibitor) in addition to 100 µM ʟ-Trp.

The reaction was carried for 60 min at 37 °C and stopped with 50 µl of 30% (w/v) TCA. The samples were next incubated for 15 min at 65 °C to convert N-formylkynurenine to Kyn and centrifuged (14,000 × *g*, 5 min, 4 °C). The obtained supernatant was transferred into a chromatographic insert vial and analysed by both HPLC-DAD and UHPLC-MS/MS (the reference method).

### 2.5. Measuring of ʟ-kynurenine by HPLC-DAD and IDO1 activity calculation

The chromatographic separation was achieved with a mobile phase composed of solvent A: 10 mM ammonium acetate in water (pH adjusted to 4.0 ± 0.05 with glacial acetic acid) and solvent B: 100% methanol. The following gradient program was applied: 0–17 min – 0% B; 17–20 min – 0–5% B; 20–30 min – 5–20% B, 30–35 min – 20–30% B; 35–40 min – 30–60% B; 40–45 min – 60–0% B; 45–50 min – 0%B. The flow rate was set at 0.5 ml/min and 5 µl (from SK-OV-3) or 50 µl (from MDA-MB-231) of samples were injected on the column. ʟ-Kyn (monitored at 360 nm) appeared at retention time *t_r_* = 15.5 min. The signal for ʟ-Trp was monitored at 284 nm at the *t_r_* about 29.8 min to control chromatographic performance. The column temperature was set at 40 °C. The peak area of ʟ-Kyn was determined using the Agilent ChemStation software v.B.04.02 based on the standard curve obtained in presence of a sample matrix. IDO1 activity was expressed as nmol of ʟ-Kyn formed within 1 min per 1 mg of protein. Inhibition expressed as a percentage of generated ʟ-Kyn was calculated as (100 − (A/B × 100)), where A and B is the peak area of ʟ-Kyn measured in the presence or absence of inhibitor (ʟ-1MT), respectively.

### 2.6. UHPLC-MS/MS analysis

The mobile phase consisted of solvent A (5 mM ammonium acetate in water) and solvent B (100% methanol). The separation was achieved within 6 min run applying for the following gradient program: 0–2 min – 5–50% B; 2–3 min – 50–90% B, and from 3 min – from 5% B. The flow rate was set at 0.25 ml/min and column temperature at 40 °C. The injection volume was 5 µl and the analyte was detected at *t_r_* = 1.41 min. ʟ-Kyn detection was performed by the multiple reaction monitoring (MRM) mode. Transitions of ions of *m/z* 209.0 to ion of *m/z* 192.0 (quantifier, collision energy, CE 8 eV) and to ion of *m/z* 174.0 (qualifier, CE 10 eV). The electrospray source conditions were: fragmentor 100 V, gas temperature 300 °C, gas flow 5 L/min, nebuliser gas 35 psi, sheath gas temperature 300 °C, sheath gas flow 7 L/min, capillary voltage 3500 V. For quantification, a calibration curve was constructed in the range from 0.022 to 8.6 µM for standards prepared in the IDO1 activity reaction mixture (linear regression equation was *y* = 114788*x* + 3374.4, coefficient of determination (*r*^2^): 0.9985). If necessary, samples were diluted 10 times before being injected into the column. All measurements were done in triplicates.

### 2.7. Total protein content estimation

The protein concentration of the cell extracts was measured using Bradford’s protocol^39^ with BSA (bovine serum albumin) standard solution (1.23 µg/ml) for calibration curve following the procedure described earlier^36^^,^^37^.

### 2.8. Validation of the HPLC-DAD method

#### 2.8.1. Calibration curve

The standard solutions of ʟ-Kyn were prepared in IDO1 activity reaction mixture to obtain a calibration range from 0.043–8.6 μM. All calibration points were run in triplicates. Measurements were performed on 3 different days within 1 month. The calibration curve was established by plotting the mean ʟ-Kyn peak area versus the concentration of the analyte. The lowest detected (limit of detection, LOD) and quantified (limit of quantification, LOQ) amount of ʟ-Kyn were determined using Equations (1) and (2), respectively:

(1) $$\mathrm{LOD}\;(\mu M)=\frac{ʟ-\text{Kyn concentration}}{\text{Average S}/N}\times \;3$$

(2) $$\mathrm{LOQ}\;(\mu M)=\frac{ʟ-\text{Kyn concentration}}{\text{Average S}/N}\times \;10$$

An average signal-to-noise (S/N) ratio was calculated for the spiked sample at the lowest detectable concentration (*n* = 6).

#### 2.8.2. Precision and accuracy

The precision and accuracy were determined by analysing three concentrations in the linear range corresponding to low (LOQ), medium, and high (the upper limit of quantification, ULOQ). The concentrations have been specified in Table 1. The test was performed using ʟ-Kyn standard dissolved in the IDO1 reaction mixture in addition to the sample containing cell lysate spiked with the analyte standard. The lysate from MDA-MB-231 cancer cells was used for this purpose since they generate a low level of ʟ-Kyn. Intraday precision was assessed in regard to the RSD of six sample replicates in a single day. Interday precision was expressed by the RSD of data obtained from 18 replicates of the samples over three different days. The accuracy (%) was calculated by dividing an average by a nominal concentration.

**Table 1.** Accuracy and interday precision estimated in standard medium and biological samples

	In IDO1 activity mixture			In cell lysate^a^		
	ʟ-Kyn concentration added (µM)					
	0.043	2.15	8.60	0.043^b^	2.15^b^	8.60^b^
Day 1						
Mean ± SD (µM)	0.039 ± 0.002	2.193 ± 0.017	8.822 ± 0.070	0.048 ± 0.005	2.428 ± 0.008	9.524 ± 0.029
RSD (%)	6.16	0.76	0.79	5.31	0.32	0.31
Accuracy (%)	90.60	101.98	103.74	112.67	112.95	110.74
Day 2						
Mean ± SD (µM)	0.040 ± 0.003	2.034 ± 0.007	8.586 ± 0.026	0.043 ± 0.010	1.982 ± 0.013	8.204 ± 0.023
RSD (%)	8.07	0.34	0.30	2.03	0.53	0.33
Accuracy (%)	93.27	94.59	99.84	111.03	89.92	95.40
Day 3						
Mean ± SD (µM)	0.039 ± 0.002	1.996 ± 0.007	8.256 ± 0.021	0.040 ± 0.006	2.102 ± 0.181	8.936 ± 0.353
RSD (%)	5.03	0.37	0.26	6.53	8.41	3.93
Accuracy (%)	90.39	92.84	96.00	91.94	97.77	103.91

^a^Cell lysate from MDA-MB-231 cancer cells; ^b^calculated as follows: ʟ-Kyn concentration in spiked sample (µM) – ʟ-Kyn concentration in blank sample (µM).

Standard deviation (SD) values were calculated from 6 measurements.

#### 2.8.3. Recovery and matrix effect

The recovery and matrix effect were evaluated using Equations (3) and (4), respectively. The studies were carried out using aliquots containing MDA-MD-231 cancer cells lysate and ʟ-Kyn spike at the low (0.043 µM), medium (2.15 µM) and high (8.60 µM) concentration levels. The recovery was investigated by comparing ʟ-Kyn peak area for samples spiked at the beginning of the sample preparation step (A) with the peak area obtained for samples fortified with the analyte at the final step, before injection onto column (B). The matrix effect was determined by comparing the peak area of the analyte in the IDO1 reaction mixture (without cell lysate) (C) with one obtained for the sample fortified at the final step of sample preparation (B). Due to the existence of the endogenous ʟ-Kyn in MDA-MB-231 matrix (cell lysate), the response of the blank was subtracted from the values obtained for samples A and B. All samples were run in 6 replicates.

(3) $$\mathrm{Recovery}\;(\%)=\frac{\text{Mean peak area in sample A }-\text{ Mean peak area in blank sample}}{\text{Mean peak area in sample B }-\text{ Mean peak area in blank sample}}\times \;100$$

(4) $$\text{Matrix effect }(\%)=\frac{\text{Mean peak area in sample B }-\text{ Mean peak area in blank sample}}{\text{Mean peak area in sample C}}\times \;100$$

#### 2.8.4. Selectivity

The selectivity of the herein proposed method was evaluated by analysing blank samples from different types of cancer cell lines (MDA-MB-231 and SK-OV-3) prepared as was described in sample preparation. The samples from at least 10 individual wells were run in duplicates.

#### 2.8.5. Stability

The stability of ʟ-Kyn in IDO1 activity mixture was evaluated at three different concentration levels (0.043, 2.15, and 8.60 µM). The experimental samples were kept for 24 h at room temperature in an autosampler tray. All samples were run in 6 replicates. Stability was estimated as a percentage of the concentration obtained for freshly prepared standards.

### 2.9. Statistical analysis

Data were presented as average ± standard deviation (SD). The student’s *t*-test was performed using XLSTAT 2018.5.52140 software to evaluate the accuracy of the HPLC-DAD method. Differences between IDO1 activity in control cancer cells and cells exposed to AGEs were determined using the Mann–Whitney *U* test using Statistica 13.1 software. A level of probability *p* < 0.05 was used to denote statistically significant differences between the studied groups.

## 3. Results

### 3.1. Optimisation of chromatographic separation

Preliminary studies were carried out using a mobile phase composed of 10 mM ammonium acetate in water (pH 5.0 with acetic acid, solvent A) and methanol (solvent B) with a flow rate set at 0.8 ml/min. The percentage contribution of solvent A and B in the mobile phase and gradient program were changed in order to get analyte resolution from other compounds Optimisation was evaluated by comparing the chromatograms of ʟ-Kyn standard (prepared in the IDO1 activity reaction mixture) with the samples containing MDA-MB-231 cell lysates (prepared in the IDO1 activity assay with 400 µM ʟ-Trp) and monitoring of the eluted ʟ-Kyn peak with absorbance at 225 nm.

The results showed co-elution of some endogenous compounds with ʟ-Kyn, despite a decrease in flow rate from 0.8 ml/min to 0.5 ml/min. To resolve the problem, the pH of solvent A was investigated. The pH of 10 mM ammonium acetate was reduced from 6.6 to 3.5 by adding acetic acid. Changing pH of the solvent A (the flow rate 0.5 ml/min) had a negligible effect on the retention time of ʟ-Kyn (15.5 ± 0.5 min). However, we observed a change in ʟ-Kyn resolution from other sample matrix components depending on the used pH of solvent A. The most serious interferences of closely eluting compounds on the ʟ-Kyn peak were noted at a pH of 6.60. After visual evaluation of the obtained chromatograms, 10 mM ammonium acetate with a pH of 4.0 was selected as the optimal solvent A for further experiments.

**Figure 1.**
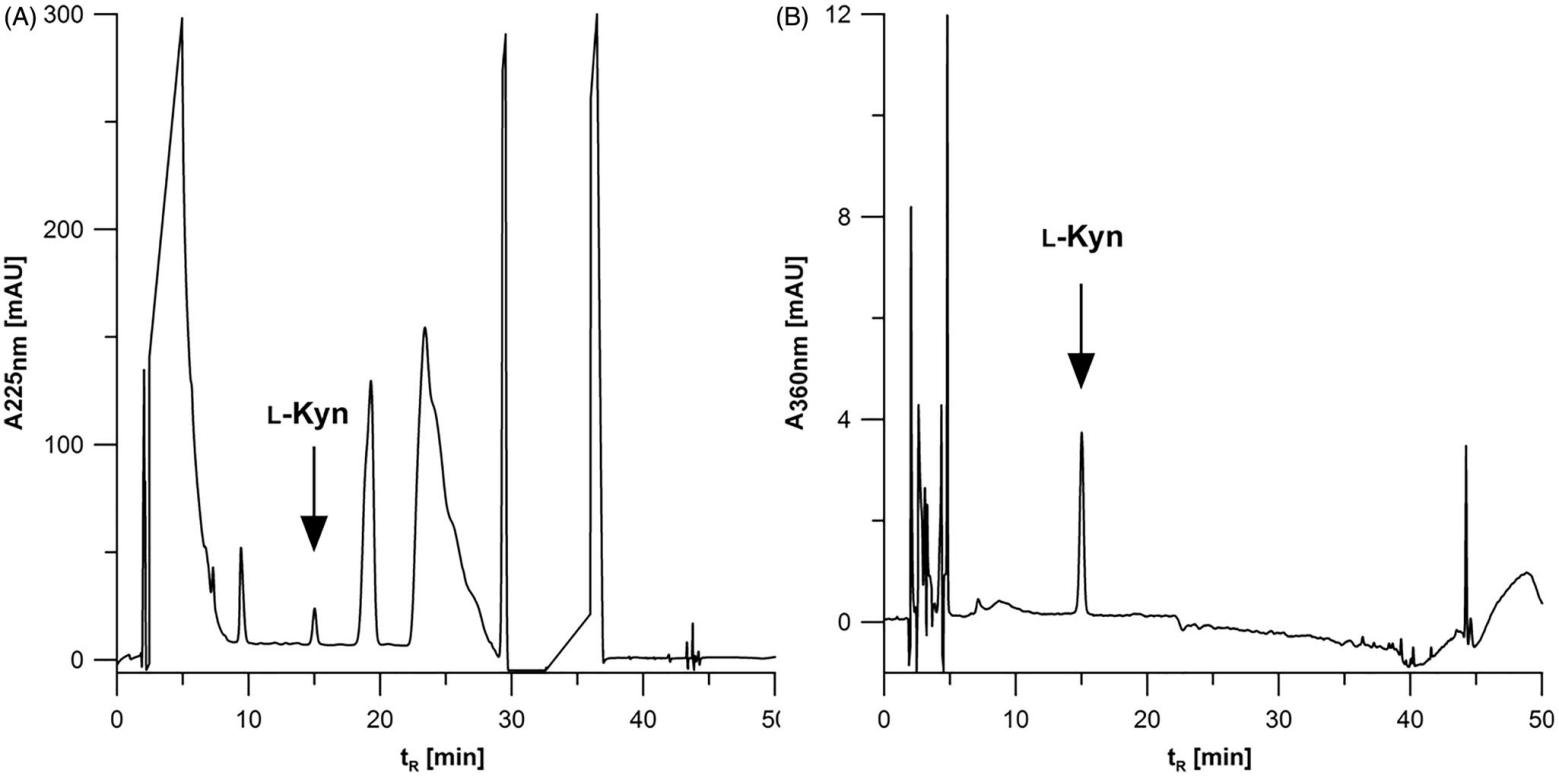
Comparison of chromatograms registered at (A) 225 and (B) 360 nm for ʟ-Kyn quantification in lysate of MDA-MB-231 cancer cells fortified with the analyte (2.15 µM). Injection volume: 50 µl.

In the next step, the effect of the monitored wavelength was also investigated. Based on literature regarding the determination of ʟ-Kyn by HPLC-UV^1^ two wavelengths at 225 and 360 nm were evaluated. The chromatograms of MDA-MB-231 cell lysate fortified with ʟ-Kyn and registered at 225 and 360 nm are shown in Figure 1. The analysis at 225 nm (Figure 1(A)) and 360 nm (Figure 1(B)) is carried out with similar resolution (*Rs*). The calculated *Rs* at 225 and 360 nm for MDA-MB-231 lysate was about 2.50 and 2.93, respectively. In the case of SK-OV-3, *Rs* reached about 2.43 and 3.73 at 225 nm and 360 nm, respectively. Although at both wavelengths the resolution is sufficient, 360 nm appears to be specific for the detection of ʟ-Kyn under the applied chromatographic conditions. ʟ-Kyn monitoring at 360 nm delivered better selectivity and is recommended when working with different cell lines to obtain accurate data.

Finally, to test the optimised conditions, the chromatograms of 2.15 µM ʟ-Kyn standard solutions (six replicates) were evaluated to determine the key chromatographic parameters. The average retention time of ʟ-Kyn was 15.52 ± 0.02 min, retention factor (*k*) was 6.659 ± 0.005, and the estimated number of theoretical plates (N) and peak asymmetry (As) were 5119 ± 67 and 1.04 ± 0.02, respectively.

### 3.2. Adjustment of the sample preparation protocol

The IDO1 activity assay was based on measuring ʟ-Kyn formation rate generated by the enzyme present in the cytoplasmic fraction of the cancer cell lysate. To initiate an enzymatic reaction a sample of cell lysate was transferred to a reaction mixture^21^^,^^30^^,^^38^^,^^40^. It contains ascorbic acid, methylene blue (a reducing system required for catalytic activity), catalase (to minimise the inhibitory effect caused by hydrogen peroxide produced by the reducing system), phosphate buffer pH 6.5 (to ensure optimal pH for ʟ-Trp oxidation) and ʟ-Trp (the enzyme-substrate). In our work, to observe the maximal activity of IDO1 enzyme, some key parameters such as the amount of cell lysate, the final volume of the reaction mixture, and concentration of substrate were optimised. The protocol was developed using the freshly collected MDA-MB-231 and SK-OV-3 cancer cell lysates that significantly differ in a basal level of the enzyme activity.

At first, we tested different amount (from 20 to 90 µl) of cell lysates added into the reaction mixture kept at 200 µl final volume in presence of 400 µM concentration of ʟ-Trp. The human cancer cells with low basal IDO1 activity (MDA-MB-231) and thus low ʟ-Kyn production were selected for optimisation. We observed that an amount of the enzyme present in 90 µl of cell lysate generated the best ʟ-Kyn signals and reducing the final volume to 150 µl further improved the method sensitivity.

**Figure 2.**
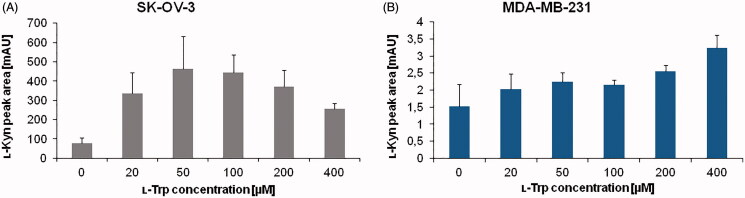
Effect of ʟ-Trp concentration ranging from 0 to 400 µM on ʟ-Kyn production by IDO1 present in (A) SK-OV-3 and (B) MDA-MB-231 cancer cells. For each experiment, lysate from 5 × 10^6^ cells were used. The values represent means of two measurements. Injection volume: 50 µl.

Next, we investigated substrate amount effect on IDO1 activity by changing the concentration of ʟ-Trp in the reaction mixture between 0 to 400 µM. The experiment was carried out in parallel with SK-OV-3 and MDA-MB-231 cells in order to identify universal conditions for different cancer cells. The peak area of ʟ-Kyn generated by cellular enzyme was assessed to determine optimal substrate concentration (Figure 2). Increasing the ʟ-Trp amount in the reaction mixture up to 50 µM resulted in enhancement of ʟ-Kyn production by SK-OV-3 cancer cells, however the ʟ-Kyn signal decreased for 200 and 400 µM (Figure 2(A)). In the case of MDA-MB-231 cells, an impact of ʟ-Trp concentration on ʟ-Kyn production by IDO1 activity was significantly lower (Figure 2(B)), although a slight increase of ʟ-Kyn signal was observed from 20 to 400 µM (Figure 2(B)). It shows the importance of using the optimal substrate concentration to yield accurate results, in particular in cells with high IDO1 activity like SK-OV-3. This is due to reaching an optimal enzyme velocity in presence of the balanced ratio of substrate to enzyme. After a certain optimal substrate concentration, an enzyme becomes saturated and a further increase in ʟ-Trp amount decreases enzyme activity. This substrate-inhibition was proposed to be an aftereffect of ʟ-Trp binding in an inhibitory site of the enzyme^41^^,^^42^. We concluded based on the obtained results that100 µM ʟ-Trp is the optimal concentration and it was chosen for further experiments.

Finally, an impact of cell freezing before performing IDO1 assay was also evaluated. After cells were harvested, one portion was immediately subjected to IDO1 activity measurements using the optimal reaction conditions. The second part was frozen at −70 °C until use. We observed a significant decrease (about 65%) of ʟ-Kyn production in previously frozen SK-OV-3 cells compared to the fresh ones. In the case of MDA-MB-231 cells, the loss was smaller (about 22%), probably due to initially low enzyme activity. The results clearly indicate the importance of using freshly collected cells to achieve a proper estimation of IDO1 activity in cancer cells.

### 3.3. Inhibition studies

The inhibition of IDO1 activity was investigated in order to confirm that the developed method can serve for specific IDO1 enzyme activity measurements. A wide range of IDO1 inhibitors have already been identified, including compounds such as LM10 (6-Fluoro-3-[(1E)-2-(2H-tetrazol-5-yl)ethenyl]-1H-indole)^43^, INCB024360 ((E)-N′-(3-bromo-4-fluorophenyl)-N-hydroxy-4-((2-(sulfamoylamino)ethyl)amino)-1,2,5-oxadiazole-3-carboximidamide)^44^, β-(3-benzofuranyl)-ᴅʟ-alanine, β-[3-benzo(b)thienyl]-ᴅʟ-alanine^45^, Exiguamine A, MTH-Trp, 1MT (including ʟ- and ᴅ- isomers), and many other^12^. Several studies confirmed that 1-Methyl-ʟ-tryptophan (ʟ-1MT) abrogated IDO1 activity^46^^,^^47^, thus it was chosen in this work to confirm assay applicability in the assessment of enzyme activity.

**Figure 3.**
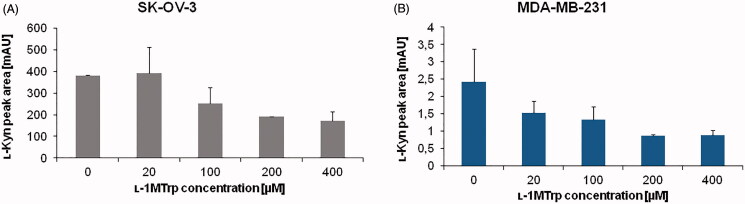
Effect of ʟ-1MT concentration on IDO1 inhibition. ʟ-Kyn peak area for (A) SK-OV-3 and (B) MDA-MB-231 cancer cells was measured. The IDO1 activity mixture contained from 0 to 400 µM 1-MT (inhibitor) in addition to100 µM ʟ-Trp (substrate). Other conditions were the same as specified for Figure 2.

The IDO1 inhibition was studied using 0–400 µM ʟ-1MT and constant amount of ʟ-Trp (100 µM) in the reaction medium (Figure 3). In the SK-OV-3 cells, inhibition of IDO1 activity was observed for ʟ-1MT of 100 µM and more. The observed percentage of IDO1 inhibition reached 33.73, 50.19, and 55.22% for 100, 200, and 400 µM ʟ-1MT, respectively). In the case of MDA-MB-231 cells 36.96% decrease of ʟ-Kyn signal was obtained for inhibitor concentration of 20 µM. Applying a higher concentration of ʟ-1MT resulted in 44.66, 64.19, and 63.48% of initial ʟ-Kyn signal for 100, 200, and 400 µM inhibitor, respectively). The observed decrease in the amount of ʟ-Kyn (increased inhibition) confirms method specificity towards IDO1 activity.

### 3.4. Analytical method validation

#### 3.4.1. Calibration curve

The method showed good linearity in standards tested for three different days at seven concentrations of ʟ-Kyn from 0.043–8.6 µM. The mean linear regression equation of the standard curve was *y* = 30.787*x* + 0.192 (standard deviation for slope and intercept were ±2.663 and 0.954 (*n* = 3), respectively) with an excellent coefficient of determination (*r*^2^) of 0.9999. The calculated LOD and LOQ of ʟ-Kyn were 12.9 and 43.0 nM, respectively. It corresponds to 0.66 and 2.20 pmol of Kyn, respectively.

#### 3.4.2. Precision and accuracy

The accuracy and intraday precision results were collected in Table 1. The Kyn quantification accuracy estimated in the IDO1 reaction mixture and in samples containing cell lysate ranged from 90.39 to 103.74% and from 89.92 to 112.95%, respectively. All results were within ± 15% of nominal concentration, which meets the internationally accepted criteria for method validation^48^. Intraday precision was 0.26–8.07% and 0.31–8.41% for the IDO1 reaction mixture and samples with cell lysate, respectively. Interday precision (*n* = 18) in the standard medium was 6.59, 4.26, and 3.27% for 0.043, 2.15, and 8.60 µM ʟ-Kyn, respectively. Interday precision (*n* = 18) estimated in presence of the sample matrix was 10.95, 11.68, and 7.44% for 0.043, 2.15, and 8.60 µM ʟ-Kyn, respectively. All CV values of intraday and interday precision were in the acceptable range (less than 15%). These results indicated the reliability of the optimised HPLC-DAD method for ʟ-Kyn determination employed in IDO1 activity measurement in cancer cells.

#### 3.4.3. Recovery and matrix effect

The assay showed analyte recovery in cancer cell matrix at 93.72, 103.25, and 92.13% for 0.043, 2.15, and 8.60 µM ʟ-Kyn, respectively. Furthermore, no serious enhancement of the analyte signal in presence of cell lysate was observed for any investigated ʟ-Kyn concentration levels. The estimated matrix effect was at the level of 110.29, 110.44 and 106.67% for 0.043, 2.15, and 8.60 µM ʟ-Kyn, respectively.

#### 3.4.4. Selectivity

**Figure 4.**
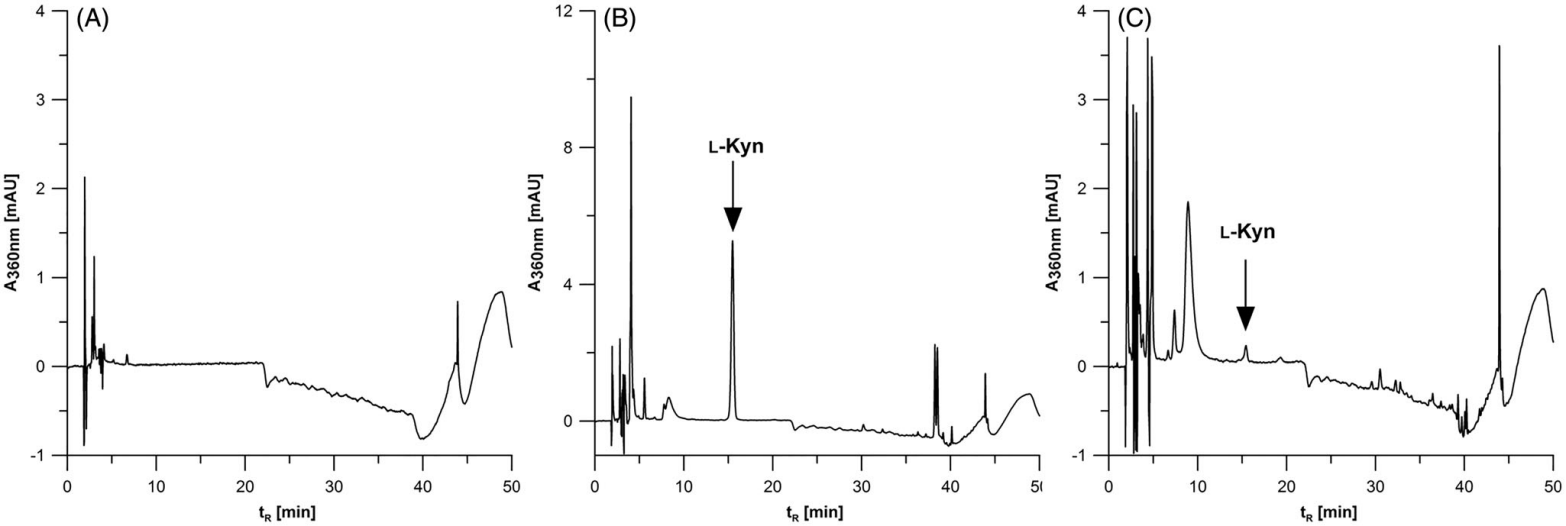
The HPLC-DAD results for IDO1 activity mixture alone (A) and in presence of cancer cell lysate fromSK-OV-3 (B) and MDA-MB-231 (C).

The typical chromatogram obtained during ʟ-Kyn determination by the HPLC-DAD method in the blank samples of the IDO1 reaction mixture and in samples containing MDA-MB-231 or SK-OV-3 cell lysates (Figure 4) indicates no interference with the analyte signal associated with other sample components. It confirms that our method is selective for the determination of ʟ-Kyn production by IDO1 enzyme in cancer cells.

#### 3.4.5. Stability

The working solutions of ʟ-Kyn prepared in the IDO1 reaction mixture were stable for 24 h when stored in an autosampler at room temperature. For each concentration level tested, no more than 10% degradation of the analyte was noted (90.23, 98.32, and 97.71% for 0.043, 2.15, and 8.60 µM ʟ-Kyn, respectively).

### 3.5. Confirmation of the method accuracy by the reference method

The proposed HPLC-DAD method for estimation of IDO1 activity in human cancer cells was verified for accuracy using UHPLC-MS/MS as the reference method. The basal IDO1 activity in MDA-MB-231 and SK-OV-3 cells was measured in samples prepared according to the protocol described in the “Materials and methods” section. As excepted based on previous HPLC-DAD measurements, IDO1 activity estimated by UHPLC-MS/MS (run in triplicates) in SK-OV-3 cancer cells were much higher compared to MDA-MB-231 cells. In four different samples of SK-OV-3 cells activity range was from 3.4921 to 4.3085 nmol ʟ-Kyn/min/mg protein, while in MDA-MB-231 cells IDO1 activity was as low as 0.0051 to 0.0146 nmol ʟ-Kyn/min/mg protein (Table 2). A similar observation was true for IDO1 activity measured by HPLC-DAD method. The comparison of results obtained by two alternative methods shown in Table 2 indicates high convergence. There were no statistically important differences between results obtained by HPLC-DAD and UHPLC-MS/MS methods calculated with *t-*test (all *p*-values > 0.05).

**Table 2.** IDO1 activity in human cancer cells

Sample	HPLC-DAD	LC-MS/MS	*p*-Value
	IDO1 activity ± SD (nmol/mg per min)		
MDA-MB-231			
#1	0.0090 ± 0.0016	0.0095 ± 0.0002	0.3160
#2	0.0120 ± 0.0031	0.0146 ± 0.0038	0.2800
#3	0.0080 ± 0.0011	0.0075 ± 0.0003	0.7296
#4	0.0060 ± 0.0002	0.0051 ± 0.0004	0.9680
#5	0.0116 ± 0.0028	0.0135 ± 0.0010	0.2010
#6	0.0114 ± 0.0026	0.0124 ± 0.0003	0.3014
SK-OV-3			
#7	4.4199 ± 0.3793	3.7689 ± 0.0911	0.9488
#8	3.5507 ± 0.3180	3.4921 ± 0.1692	0.5909
#9	5.0456 ± 0.2751	4.3200 ± 0.1314	0.9704
#10	5.1489 ± 0.4440	4.3085 ± 0.0899	0.9237
#11	4.1634 ± 0.7006	3.9443 ± 0.3253	0.6577
#12	4.3314 ± 1.4730	3.9034 ± 0.3076	0.6642

SD: standard deviation from 3 measurements.

**Figure 5.**
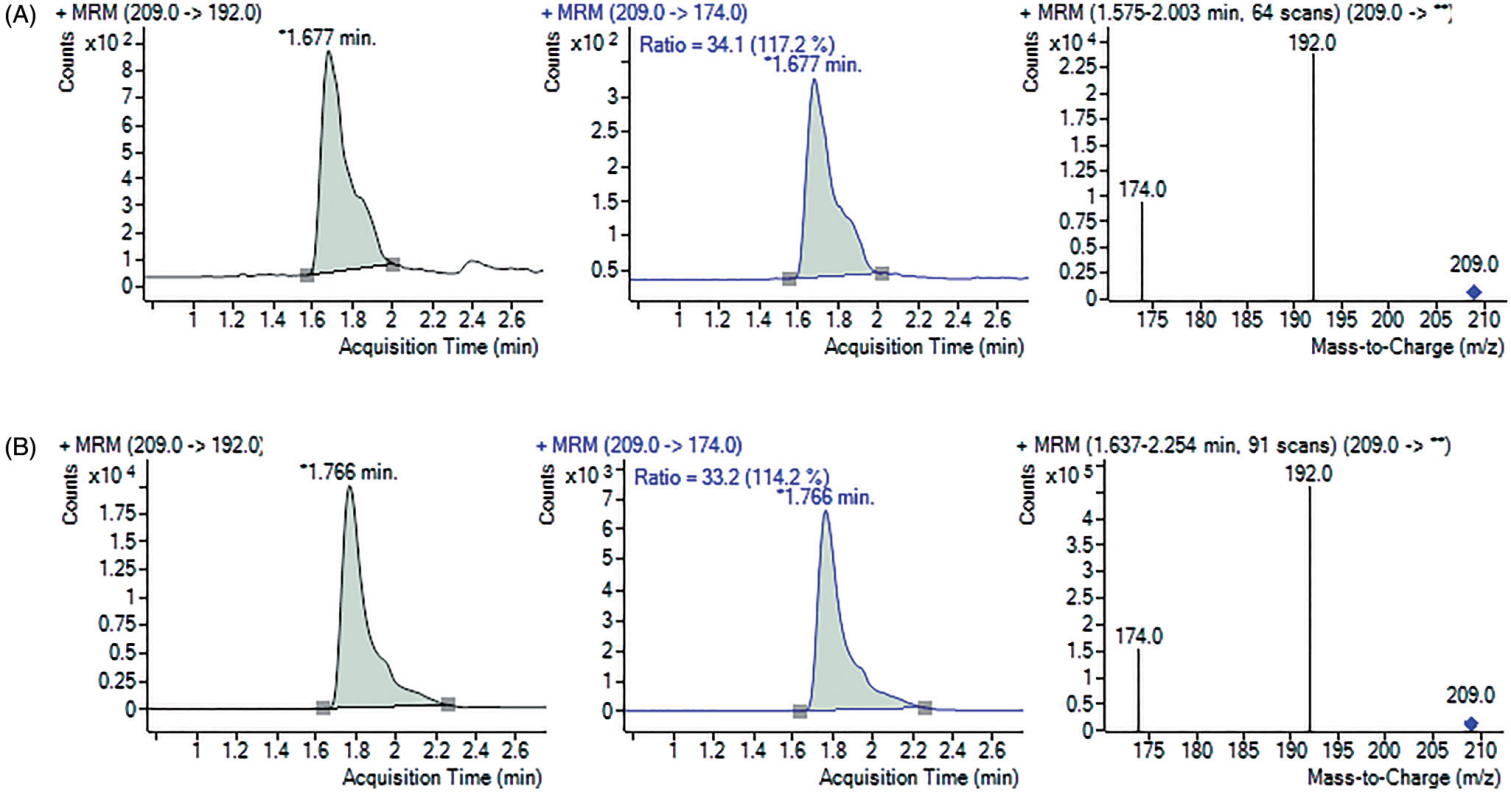
UHPLC-MS/MS chromatograms and spectra of samples for MDA-MB-231 (A) and SK-OV-3 (B) cancer cells. Multiple reaction monitoring (MRM) transitions of ions of *m/z* 209.0 to ion of *m/z* 192.0 (quantifier) and to ion of *m/z* 174.0 (qualifier) were selected for ʟ-Kyn determination.

The representative chromatograms obtained during HPLC-DAD determination of ʟ-Kyn are presented in Figure 4(A) for the blank IDO1 reaction mixture, MDA-MB-231 (Figure 4(B)), and SK-OV-3 cell lysates (Figure 4(C)). In comparison, chromatograms and spectra showing the characteristic ʟ-Kyn molecular and fragmentation ions (*m/z* 209, 192, 174) that were recorded using UHPLC-MS/MS are shown for MDA-MB-231 (Figure 5(A)) and SK-OV-3 cells (Figure 5(B)). The results confirm that herein proposed HPLC-DAD protocol for measurement of ʟ-Kyn produced by IDO1 in cancer cells provides accurate and reliable data.

### 3.6. IDO1 activity measurement in human cancer cells treated with AGEs

**Figure 6.**
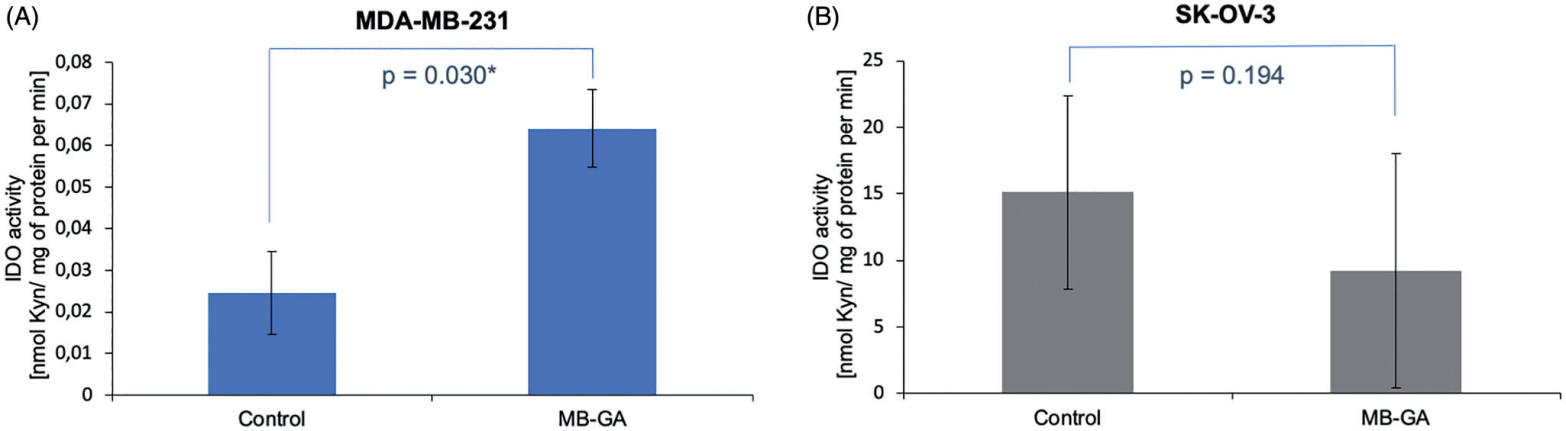
Cell-dependent effect of AGEs on IDO1 activity measured in MDA-MB-231 (A) and SK-OV-3 (B) cancer cells. The activity was assessed in lysate obtained from cancer cells cultured in complete medium (Control) or exposed for 48 h to 100 µg/ml of glycation products (AGEs) derived from glycolaldehyde (MB-GA). IDO1 activity was expressed by measuring of ʟ-Kyn amount generated by mg of cell lysate proteins (nmol/mg) during 1 min. Average activity rate was calculated from *n* = 4 for each group.

To demonstrate the applicability of the method, we determined IDO1 activity in MDA-MB-231 and SK-OV-3 cells cultured in complete medium or exposed to 100 µg/ml of AGEs (MB-GA). Sample from each individual well was injected once onto HPLC-DAD system. The results show a different level of IDO1 activity in MDA-MB-231 (Figure 6(A), *n* = 4) and SK-OV-3 (Figure 6(B), *n* = 4) cells with an average basal level of 0.025 and 15.113 nmol ʟ-Kyn/min/mg protein, respectively. Interestingly, we were able to detect changes in enzyme activity upon cell treatment with100 µg/ml of AGEs resulting in IDO1 activity increase in MDA-MB-231 cells (*n* = 4) up to an average of 0.064 nmol ʟ-Kyn/min/mg protein and the difference was statistically significant (*p* < 0.05). In contrast, in the SK-OV-3 cells presenting about 100-fold higher basal IDO1 activity it was decreased down to an average of 9.209 nmol ʟ-Kyn/min/mg protein (*n* = 4) upon treatment with 100 µg/ml AGEs (Figure 6(B)), but the observed change was not statistically significant (*p* > 0.05).

## 4. Discussion

Breast and ovarian cancer are the most common cancer type in women that makes it an important subject of research. Identification of novel therapeutic targets includes a study on the role of IDO1 in immune evasion and tumour growth^49^. High expression of IDO1 has been found in a wide range of cancer cells, including breast^50^, ovarian, lung^32^, pancreatic^51^, and liver^52^. IDO1 contributes to the development of an immunosuppressive microenvironment by the degradation of the essential amino acid tryptophan and formation of cytotoxic KP metabolites, such as kynurenine, 3-hydroxykynurenine, 3-hydroxyanthranilic acid, and quinolinic acid^32^^,^^53^. Suppression of antitumor response by IDO1 makes it an interesting therapeutic target. Methods allowing detection of IDO1 activity are in demand similar to appropriate models to test novel IDO1 inhibitors. The *in vitro* cell culture models of the established lines or primary cell cultures for decades represent a leading experimental approach at the early stages of cancer research^54^. Therefore, we aimed to develop a reliable and validated protocol for measuring of IDO1 activity in the popular ovarian (SK-OV-3) and breast cancer cells (MDA-MB-231). The selected here cancer lines previously showed IDO1 expression^32^^,^^33^. As we have was shown in Table 2, these cancer cells significantly differ in basal enzyme activity (SK-OV-3 cells show much higher IDO1 activity compared to MDA-MB-231 cells). The selection of the research material was intended in order to set the most universal conditions for IDO1 activity assay. Thus, this method could easily be adapted for other cancer cell lines. The rate of ʟ-Kyn production by the proteins obtained from cancer cells was utilised to measure enzyme activity. ʟ-Kyn level in samples was determined by HPLC-DAD. The developed protocol presents several advantages over the previously reported methods, that is, using a relatively simple and cost-efficient HPLC-DAD system, full validation, and better detection limits.

Unlike reported by others, our method was validated for linearity, the limit of detection, the limit of quantification, accuracy, precision, recovery, matrix effect and yielded reliable and reproducible analytical protocol for IDO1 activity measurement in different cancer cells and a wide range of IDO1 activity. In this study, ʟ-Kyn determination was presented over the concentration range of 43.0 nM to 8.6 µM (0.66 and 2.20 pmol) and yields to IDO1 activity even at a level of 0.006 nmol/mg per min. The protocol can also be used for samples containing a higher concentration of analyte exceeding the ULOQ after sample dilution or reduction of injection volume to fit the linear range of the method. On the other hand, for cells with very low IDO1 activity, ʟ-Kyn quantification may be possible by increasing the number of collected cells (>0.3 × 10^6^ cells per sample). Of note, the achieved method LOD (12.9 nM) was lower compared to other HPLC-based approaches previously used to determine IDO1 activity in cells^23^. The validation included also intraday and interday precision values (estimated in presence of sample matrix) that were below 10%. Furthermore, the method delivers satisfactory accuracy (within ± 10% of nominal concentration) and analytical recoveries (92.13–103.25%). All experiments were carried out using the same analytical column (more than 4000 runs). In routine use, one guard column allowed us to make approximately 450 runs before a replacement was required.

The technical difficulty of our method is associated with time-consuming sample preparation for the HPLC analysis. Nevertheless, this effort is typical for all protocols dealing with IDO1 activity measurements in cells.

Importantly, the described protocol was applied for IDO1 enzymatic activity estimation in two different human cancer cells (ovarian: SK-OV-3 and breast: MDA-MB-231). We found that basal ʟ-Kyn production in tested human breast cancer cells is much lower than in ovarian ones (Table 2). The estimated IDO1 activity in MDA-MB-231 and SK-OV-3 cancer cells were 0.006–0.039 and 3.551–25.874 nmol ʟ-Kyn/min/mg protein, respectively. These results clearly demonstrate that different types of cancer cells may significantly differ in IDO1 activity. This is of great importance when choosing an appropriate cellular model that is, for studying IDO1 as a therapeutic target. Relying only on positive results of protein expression measured by RT-PCR or immunostaining might not show real enzyme activity like in studied MDA-MB-231 cells that were considered IDO1 positive in other report^31^, however, enzymatic activity is of magnitude different comparing with other IDO1 positive cells, that is, SK-OV-3 when measuring a rate of Kyn generation from Trp substrate (Table 2). Our results for MDA-MB-231 cells were lower than those reported by Travers and others, however, of similar magnitude (0.12 ± 0.05 nmol ʟ-Kyn/min/mg protein)^55^. The low basal level of IDO1 expression in MDA-MB-231 cells was also observed in some other studies, although results were obtained using RT-PCR analysis or Western Blotting^31^^,^^56^^,^^57^.

Tumour cells expressing IDO1 were found in prostatic, endometrial, bladder, colorectal, pancreatic gastric, cervical, breast carcinomas, and also in many other types of cancer^5^. Among them, high expression of this enzyme was found in some malignant tumours, such as colorectal, endometrial, lung, ovarian, and renal cancer^58^. There are differences in IDO1 expression and activity between cells derived from different types of cancer, moreover, overexpression of IDO1 in some cancer tissues depends on tumour invasiveness and progression^59^. In accordance with results reported in previous research papers, we found that basal ʟ-Kyn production in the SK-OV-3 cell line is on a high level. Unfortunately, comparison of the exact amount of ʟ-Kyn produced by IDO1 in these cells is difficult due to a different approach (measuring the concentration of ʟ-Kyn released by cells into culture medium)^36^^,^^60^, sample preparation and amount of sample applied on the column^32^^,^^47^ or different method of the assay (colorimetric determination using Ehrlich’s reagent)^61^^,^^62^.

## 5. Concluding remarks

Tryptophan catabolism via kynurenine pathway has been linked to several metabolic, oncologic, and mental health disorders. Therefore, there is a clinical need for accurate estimation of tryptophan metabolism under pathological conditions. This work outlines the development of the analytical protocol for direct assessing an enzymatic activity of IDO1 that is the rate-limiting enzyme initiating tryptophan depletion. The herein proposed HPLC-DAD method was thoroughly validated for accurate, precise analyte measurement with high reproducibility. Our methodology for IDO1 activity estimation can be an important clinical tool to study the impact of tryptophan metabolism in human cancer cells. The method can be also useful for selecting potential IDO1 inhibitors or investigating the effects of different drugs that are considered for therapy.
